# Understanding Low-Acuity Visits to the Pediatric Emergency Department

**DOI:** 10.1371/journal.pone.0128927

**Published:** 2015-06-17

**Authors:** Ken J. Farion, Megan Wright, Roger Zemek, Gina Neto, Anna Karwowska, Sandra Tse, Sarah Reid, Mona Jabbour, Stephanie Poirier, Katherine A. Moreau, Nicholas Barrowman

**Affiliations:** 1 Departments of Pediatrics, University of Ottawa, Ottawa, Ontario, Canada; 2 Department of Emergency Medicine, University of Ottawa, Ottawa, Ontario, Canada; 3 Emergency Department, Children’s Hospital of Eastern Ontario, Ottawa, Ontario, Canada; 4 Children’s Hospital of Eastern Ontario Research Institute, Ottawa, Ontario, Canada; 5 Clincial Research Unit, Children’s Hospital of Eastern Ontario Research Institute, Ottawa, Ontario, Canada; University of Colorado Denver, UNITED STATES

## Abstract

**Background:**

Canadian pediatric emergency department visits are increasing, with a disproportionate increase in low-acuity visits locally (33% of volume in 2008-09, 41% in 2011-12). We sought to understand: 1) presentation patterns and resource implications; 2) parents’ perceptions and motivations; and 3) alternate health care options considered prior to presenting with low-acuity problems.

**Methods:**

We conducted a prospective cohort study at our tertiary pediatric emergency department serving two provinces to explore differences between patients with and without a primary care provider. During four, 2-week study periods over 1 year, parents of low-acuity visits received an anonymous survey. Presentation times, interventions, diagnoses and dispositions were captured on a data collection form linked to the survey by study number.

**Results:**

Parents completed 2,443 surveys (74.1% response rate), with survey-data collection form pairs available for 2,146 visits. Overall, 89.7% of respondents had a primary care provider; 68% were family physicians. Surprisingly, 40% of visits occurred during weekday office hours and 27.3% occurred within 4 hours of symptom onset; 67.5% of those early presenters were for injuries. Few parents sought care from their primary care provider (25%), health information line (20.7%), or urgent care clinic (18.5%); 36% reported that they believed their child’s problem required the emergency department. Forty-five percent required only a history, physical exam and reassurance; only 11% required an intervention not available in an office setting. Patients without a primary care provider were significantly more likely to present during weekday office hours (*p* = 0.003), have longer symptom duration (*p*<0.001), and not know of other options (*p* = 0.001).

**Conclusions:**

Many parents seek pediatric emergency department care for low-acuity problems despite their child having a primary care provider. Ensuring timely access to these providers may help reduce pediatric emergency department overuse. Educational initiatives should inform parents about low-acuity problems and where appropriate care can/should be accessed.

## Introduction

Canada’s publicly funded health care system is frequently criticized for being one of the most expensive[1] among developed nations, yet has the poorest primary care access and wait-times for many services[2]. As a result, emergency departments have become a barometer of these access issues and the health care system in general[3]. Each province uniquely manages the health system delivery for their own population, leading to significant regional variability for access metrics[4].

In 2010–11, Canadians made almost 16 million visits to an emergency department, with 48% of those visits triaged as low-acuity[5]. Low-acuity visits make up a similar proportion of emergency department visits by pediatric patients; 42% of visits to Ontario tertiary care pediatric emergency departments during 2005–08 were low-acuity[6]. Pediatric low-acuity emergency department visits may occur for many reasons, including a lack of appropriate alternatives, parental over-estimation of the problem severity, or convenience[7,8]. While insufficient inpatient bed capacity is the most important factor leading to overcrowding in adult emergency departments[9], low-acuity visits contribute significantly more to overcrowding in the pediatric emergency department environment[10]. Overcrowding negatively impacts quality of care for both high and low-acuity patients, leads to longer wait-times and lower patient/parent satisfaction. Staff morale and emergency department finances may also be strained[11–14]. In response to unacceptable wait-times in the busiest emergency departments in Ontario, the Ministry of Health and Long Term Care implemented a “Pay-for-Results” program in 2008, which currently provides approximately $100 million in additional funding to the 74 busiest emergency departments in the province[15]. Each department’s success implementing and sustaining improvements on defined wait-times metrics determines the supplemental funding over the global budget envelope their hospital will receive each fiscal year. The ministry’s wait-time strategy is also dependent on significant efforts to strengthen access to primary care[16].

Many emergency departments have implemented rapid assessment areas where lower acuity illness and injury may be assessed and treated more efficiently[17]. In June 2009, our tertiary pediatric emergency department introduced a rapid assessment area known as the Ambulatory Zone, where low-acuity patients receive care from dedicated staff in a designated area. This intervention enhanced patient flow for both high and low-acuity patients and significantly reduced wait-times[18]. Subsequently, an annualized 5.7% growth in patient visits has occurred over the past 6 years. However, we have experienced a disproportionate increase in low-acuity visits (33% of volume in 2008–09, 41% in 2011–12). This increase may be an unintended consequence of the improved wait-times and clinic-type environment creating a convenient alternative to their primary care provider (“Build it and they will come!”)[19].

We sought to understand the presentation patterns and resource implications of these low-acuity visits; the parental perceptions of their child’s illness/injury and motivations to use the pediatric emergency department for care; and the alternate health care options parents considered prior to coming to the pediatric emergency department with a low-acuity problem. We hypothesized that many low-acuity patients would not have an identified primary care provider; we wished to quantify this issue. Further, we felt visits should be different between patients with and without a primary care provider, as those patients with a primary care provider would be more likely to seek care from their known provider than come to the pediatric emergency department, assuming that the extensive initiatives by the Ontario Ministry of Health and Long-term Care to improve access to primary care had been successful.

## Patients and Methods

### Study design and population

The Children's Hospital of Eastern Ontario Research Ethics Board approved this study (file number 11/147X). Informed consent was obtained from the parents accompanying the child to the Emergency Department. This consent was implied with return of the survey; an option to decline use of child's data collection form data was available.

We conducted a single-centre prospective cohort study at the Children’s Hospital of Eastern Ontario (CHEO), an academic, tertiary pediatric emergency department serving patients 0–17 years of age (2011–12 annual census 66,329). It is located in Ottawa, Ontario, which is bordered by the province of Quebec. As such, CHEO is the only pediatric centre to serve a large geographic area and pediatric population of 500,000 from two provinces. The study operated 24 hours per day during four, 2-week study periods over 1 year (November/December 2011, March 2012, May/June 2012, August/September 2012). Study weeks were not randomized, but occurred approximately quarterly to capture seasonal variability and to avoid major holiday periods when primary care offices would be closed.

Patients presenting for an unplanned low-acuity visit, as determined by triage nurses utilizing the Paediatric Canadian Triage and Acuity Scale (PaedCTAS, level 4 or 5)[20], were eligible. Examples of PaedCTAS level 4 and 5 patients are listed in Table 1. Those unable to complete the study in English or French were excluded; less than 3% of our population report not being fluent in one of these languages.

**Table 1:** Examples of common Low-Acuity (PaedCTAS level 4 and 5) Presentations.

Complaint	PaedCTAS 4 Criteria	PaedCTAS 5 Criteria
Fever	Age>36 months, stable vital signs, non-toxic appearance	Not Applicable
Nausea, Vomiting and/or Diarrhea	Stable vital signs, non-toxic appearance, no signs of dehydration but ongoing fluid loss or not tolerating oral rehydration	Chronic nausea, vomiting or diarrhea, normal vital signs
Cough/Congestion	Acute cough, no respiratory distress, stable vital signs	Chronic cough, normal vital signs
Ear Ache	Mild acute pain, non-toxic appearance	Chronic mild pain
Rash	Localized cellulitis	Localized rash/irritation
Abdominal Pain	Acute mild pain or chronic moderate pain	Chronic mild pain
Head Injury	Minor head injury, no loss of consciousness	Not applicable
Laceration	Simple laceration requires sutures/glue	Simple laceration does not require closure
Extremity Injury	Acute injury, mild pain	Non-acute injury, mild pain

PaedCTAS Paediatric Canadian Triage and Acuity Scale

The study consisted of a parental survey paired with chart abstraction of the visit to obtain patient-level resource utilization and health outcomes. The parental survey tool [see Appendix 1], consisting of 8 categorical and 2 open-ended questions, was developed *de novo* by content experts to address the study objectives. Questions addressed several domains: whether the patient had an identified primary care provider and their practice type; the chief complaint and its duration; how quickly they felt their child needed to be seen; alternative options for health care sought and why those were not adequate; parental perceptions of pediatric emergency department care in general; and other comments. The survey was written to not exceed a grade 6 reading level, pre-tested by a convenience sample of parents for clarity and readability, revised, then translated into French, with back translation for confirmation. A data collection form completed by the clinician or research assistant [see Appendix 2] captured patient demographics, presentation time (hour of the day, weekday versus weekend), time to physician assessment, time to disposition, interventions (investigations, treatments, and consultations), diagnoses and disposition.

### Procedures

The study did not alter the patient’s assessment or care. Triage nurses determined PaedCTAS levels using the custom tool within our electronic triage document in use since 2008 (Sunrise Emergency Care, v 5.0, Allscripts, Chicago, Ill.). During hospital registration, the clerk provided eligible low-acuity patients and their parents with a cover letter and survey in the language of their choice (English or French). A unique study number was assigned to both the survey and to the data collection form that was attached to the patient’s chart.

Parents were asked to complete the survey while awaiting physician assessment and return it to survey boxes throughout the department. Consent was implied with return of the survey; an option to decline use of the child’s data collection form data was available. At the conclusion of the visit, the treating physician or a research assistant completed the data collection form, which was destroyed prior to data entry if the parent had requested that the data collection form not be used.

### Outcome measures

The primary outcome was the proportion of low-acuity visits by patients with and without an identified primary care provider. Secondary outcomes included determining patterns of presentation (time of day, weekday versus weekend, chief complaint, duration of symptoms); resource implications (investigations, treatments, dispositions); parents’ perceptions of their child’s illness/injury and motivations for using the pediatric emergency department; and the alternative health care sources they considered prior to coming to the department. Sub-analyses compared domain responses controlling for the presence of a primary care provider and province of residence.

### Statistical analysis

The study was both hypothesis-testing and hypothesis-generating. Based on 2010/11 data, the annual census was 60,077 with 40% of visits being low-acuity. Thus, 923 eligible visits on average were expected in each 2-week period. We anticipated that 800 families could be approached by the registration clerk per study period. Assuming survey completion of 70%, 2240 surveys would be available for analysis, allowing estimation of proportions (conservatively assumed to be 50%) with an accuracy of at least +/- 2.1%, based on the normal approximation to the binomial distribution (i.e., the variance is given by P*(1-P)/N where P is taken to be 0.5).

Surveys and anonymized data collection forms were matched using the study number and then entered into the Statistical Package for the Social Sciences (SPSS Inc., version 18.0, Chicago, Ill.). A 10% random sample had duplicate-entry to assess data entry quality. Responses to categorical questions on the survey or data collection form where “Other” was selected were independently reviewed by two investigators to re-categorize the response into an existing option or a new category. Disagreements were resolved through discussion, or by a third investigator if necessary.

Descriptive statistics were used to summarize categorical study data. Bivariate analyses (Pearson’s Chi Square, linear-by-linear test, and Fisher’s exact test, as appropriate) were used to explore differences between groups. Two-sided p-values less than 0.05 were considered statistically significant. Kappa values were calculated for the agreement between two clinician investigators tasked with categorizing “Other” responses.

Open-ended responses were analyzed using a quantitative content analysis. Similar responses were categorized together and then frequency counts were tabulated to determine how many respondents referenced each specific category. This coding was done by a Research Coordinator and audited by one of the authors (KM), a qualitative researcher. The two individuals met to discuss any discrepancies in the coding and to adjust the analysis as required. Exemplars were chosen to describe the quantitative counts presented.

## Results

### Patient characteristics

**Fig 1:**
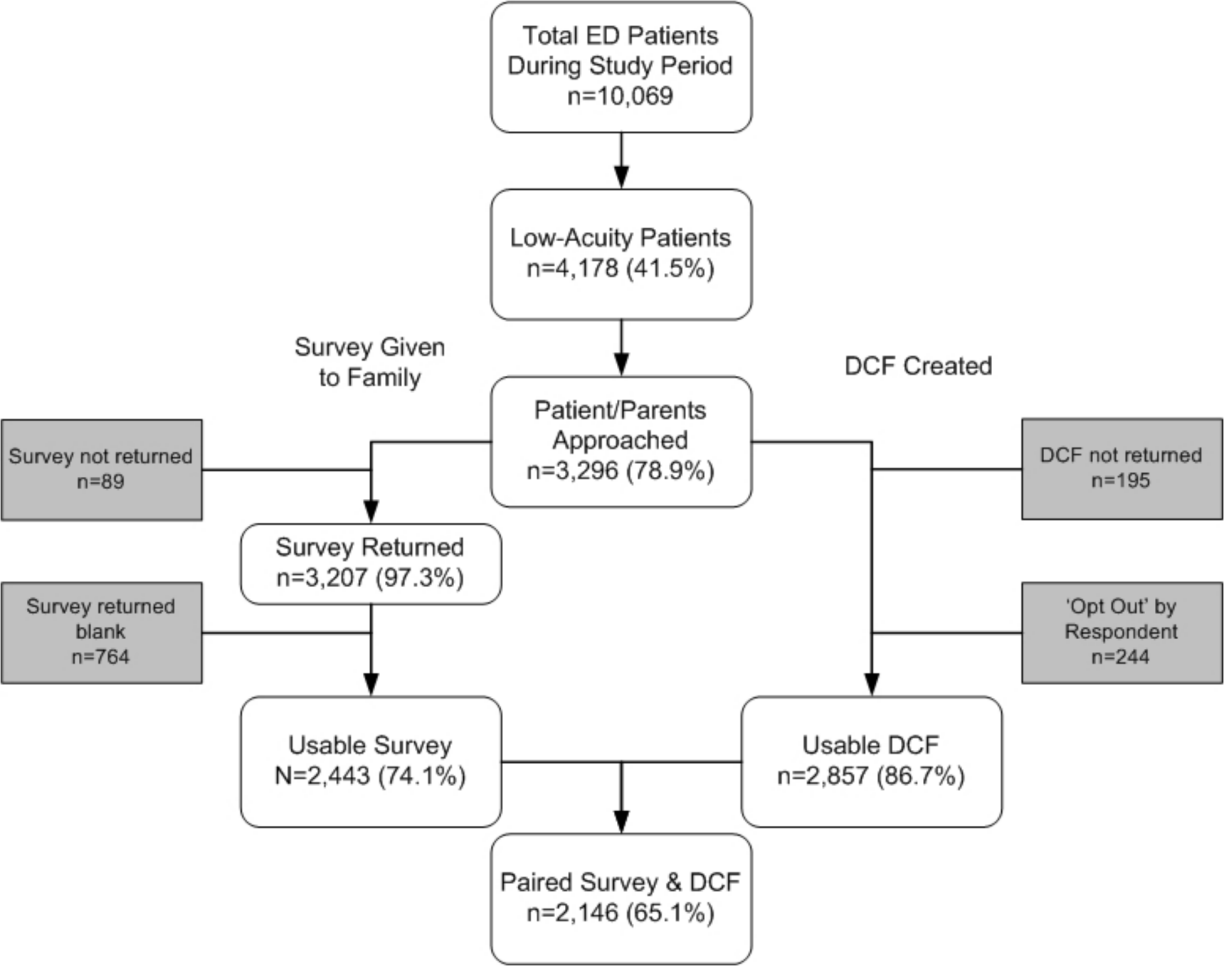
Patient recruitment and enrollment. ED, Emergency Department. DCF, Data Collection Form.

During the four, 2-week study periods, 10,069 patient visits occurred; 4,178 (41.5%) were low-acuity. A convenience sample of 3,296 eligible patients was approached (78.9% of all low-acuity visits) and completed surveys were returned for 2,443 (74.1%). Data collection forms were completed for 3,101 patients but 244 were excluded as requested, leaving 2,857 (86.7%) useable data collection forms. A total of 2,146 paired surveys and data collection forms were available for analysis, representing complete data from 65.1% of those approached. This patient recruitment and information flow is depicted in Fig 1. Agreement between clinician investigators coding “other” (free-text) responses was substantial[21] (kappa 0.75 for chief complaint and 0.65 for intervention).

Table 2 outlines the characteristics of study patients compared to all low-acuity patients and to all pediatric emergency department patients seen during the study periods. Patients from Quebec accounted for a significantly higher proportion of low-acuity patients (909 of 2,857, 31.8%) compared to the proportion of all emergency department visits by Quebec patients at the time (26%; p<0.001).

**Table 2:** Characteristics of study patients compared to all low-acuity patients and all emergency department patients during the study period.

	All Low-Acuity Study Patients with DCF data (n = 2,857)	All Low-Acuity Patients During the Study Periods(n = 4,178)	All PED Patients During the Study Periods(n = 10,069)
Age (%)			
<1 year	284 (9.9%)	374 (9.0%)	1,444 (14.3%)
1–2 years	837 (29.3%)	954 (22.8%)	2,354 (23.4%)
3–6 years	593 (20.8%)	1,243 (29.8%)	2,337 (23.2%)
7–10 years	437 (15.3%)	595 (14.2%)	1,329 (13.2%)
11–14 years	426 (14.9%)	621 (14.9%)	1,416 (14.1%)
15–18 years	280 (9.8%)	391 (9.4%)	1,189 (11.8%)
Time to MD assessment (hrs)	(n = 2,662)		
Median	1.72	1.75	1.50
90^th^ Percentile	3.40	3.45	3.32
Total Length of Stay (hrs)	(n = 2,670)		
Median	2.52	2.55	2.87
90^th^ Percentile	4.47	4.58	5.63
Disposition (%)	(n = 2,765)		
Discharged home	2,659 (96.2%)	3,978 (95.2%)	9,190 (91.3%)
Admitted to hospital	9 (0.3%)	24 (0.6%)	568 (5.6%)
Left prior to MD assessment	97 (3.5%)	176 (4.2%)	311 (3.1%)

DCF Data Collection Form

PED Pediatric Emergency Department

hrs hours

MD Medical Doctor

### Primary outcome

Within the study cohort, 1,826 of 2,036 parents (89.7%) reported having a primary care provider for their child, but this was significantly different when province of residence was considered (Ontario 1,250 of 1,328, 94.1%; Quebec 576 of 708, 81.4%; p<0.001). Respondents with a primary care provider identified them most commonly as family physicians (68.1%) or pediatricians (26.7%); 51.9% were described as working in an independent or small group practice, 29.3% were part of a family health network or team, and 18.7% of respondents were unsure of their provider’s practice type.

### Secondary outcomes

**Fig 2:**
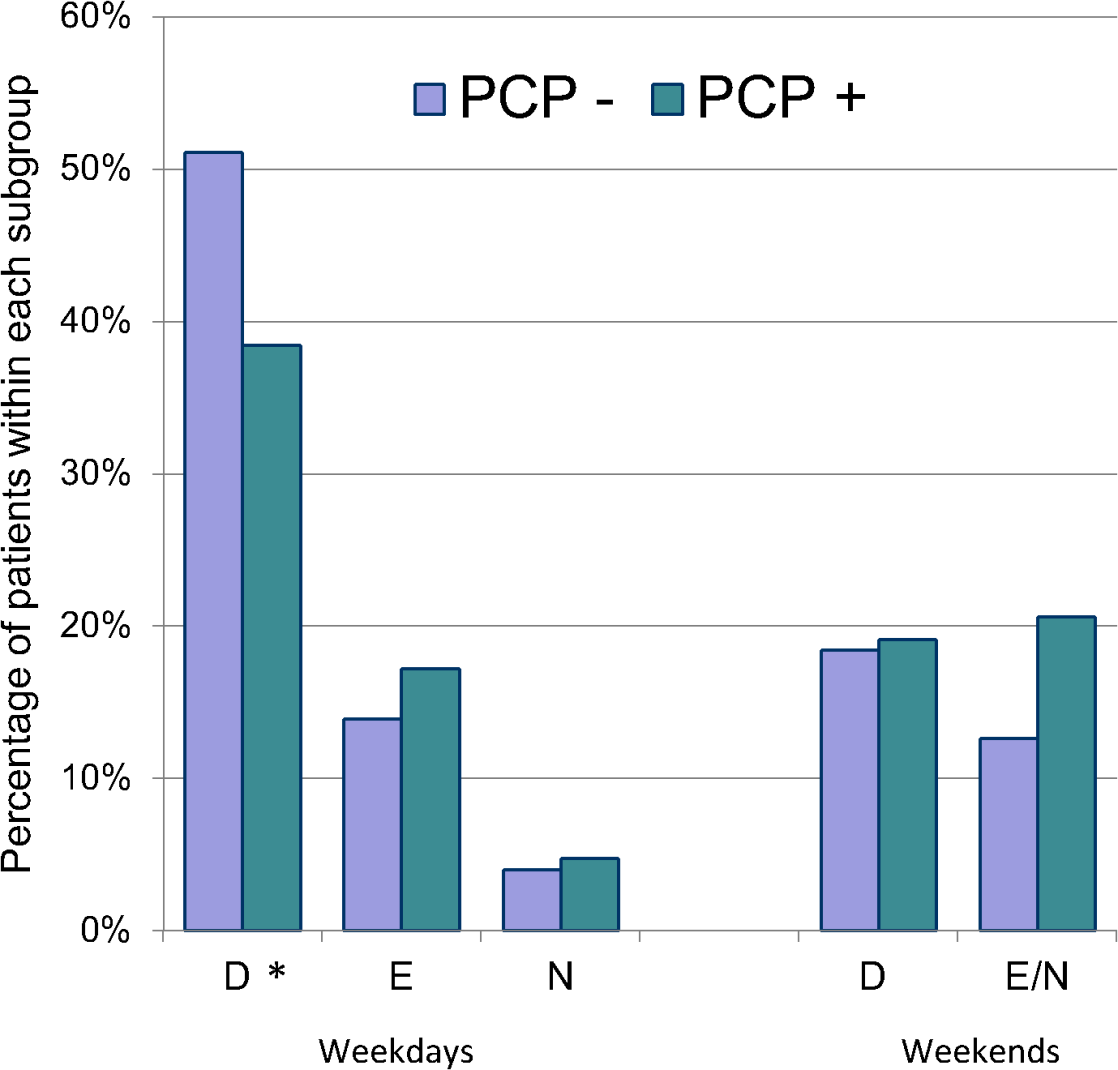
Time of Presentation to the Emergency Department. PCP-Patient does not have a primary care provider (n = 223). PCP+, Patient does have a primary care provider (n = 1,890). D, Days (08:00–17:00). E, Evenings (17:00–24:00). N, Nights (24:00–08:00). * *p* = 0.003.

Time of presentation, comparing those with and without a primary care provider, is depicted in Fig 2. Forty percent of low-acuity visits occurred during regular office hours (i.e. Monday to Friday, 8 am to 5 pm), with a significantly higher proportion amongst patients without a primary care provider (51.1%, p = 0.003 by Pearson’s chi-square test).

**Fig 3:**
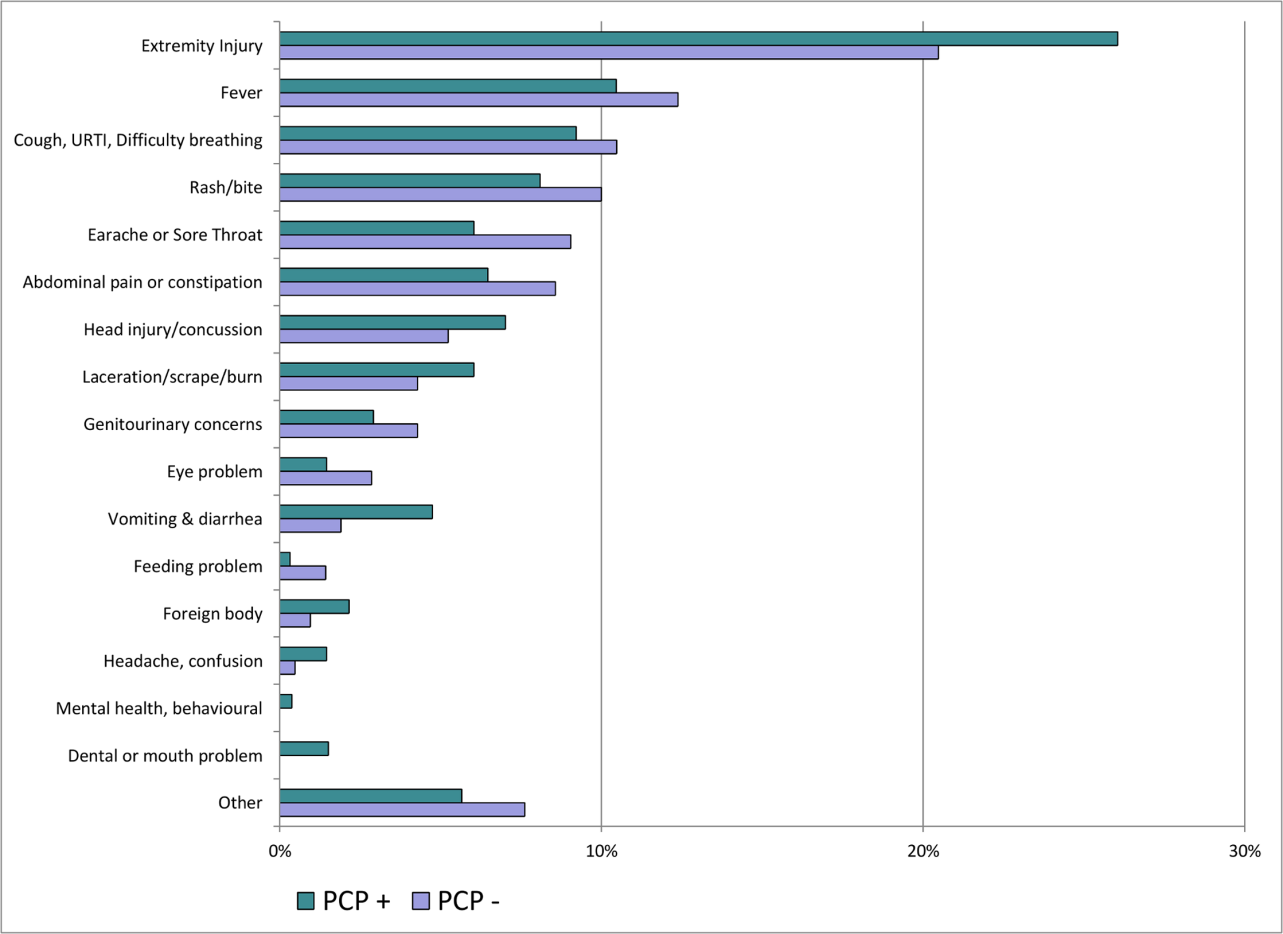
Chief complaint, as reported by the parent. PCP-, Patient does not have a primary care provider (n = 210). PCP+, Patient does have a primary care provider (n = 1,854).

The parent-reported chief complaint is depicted in Fig 3, highlighting the large proportion of minor injuries included in the cohort. When comparing patients with and without a primary care provider, there was no significant difference in the relative proportion of patients presenting with a specific chief complaint. However, when complaints were grouped, the proportion of illness-type visits was significantly higher in those without a primary care provider (69.1%) compared to those with a primary care provider (58.7%, p<0.005).

**Fig 4:**
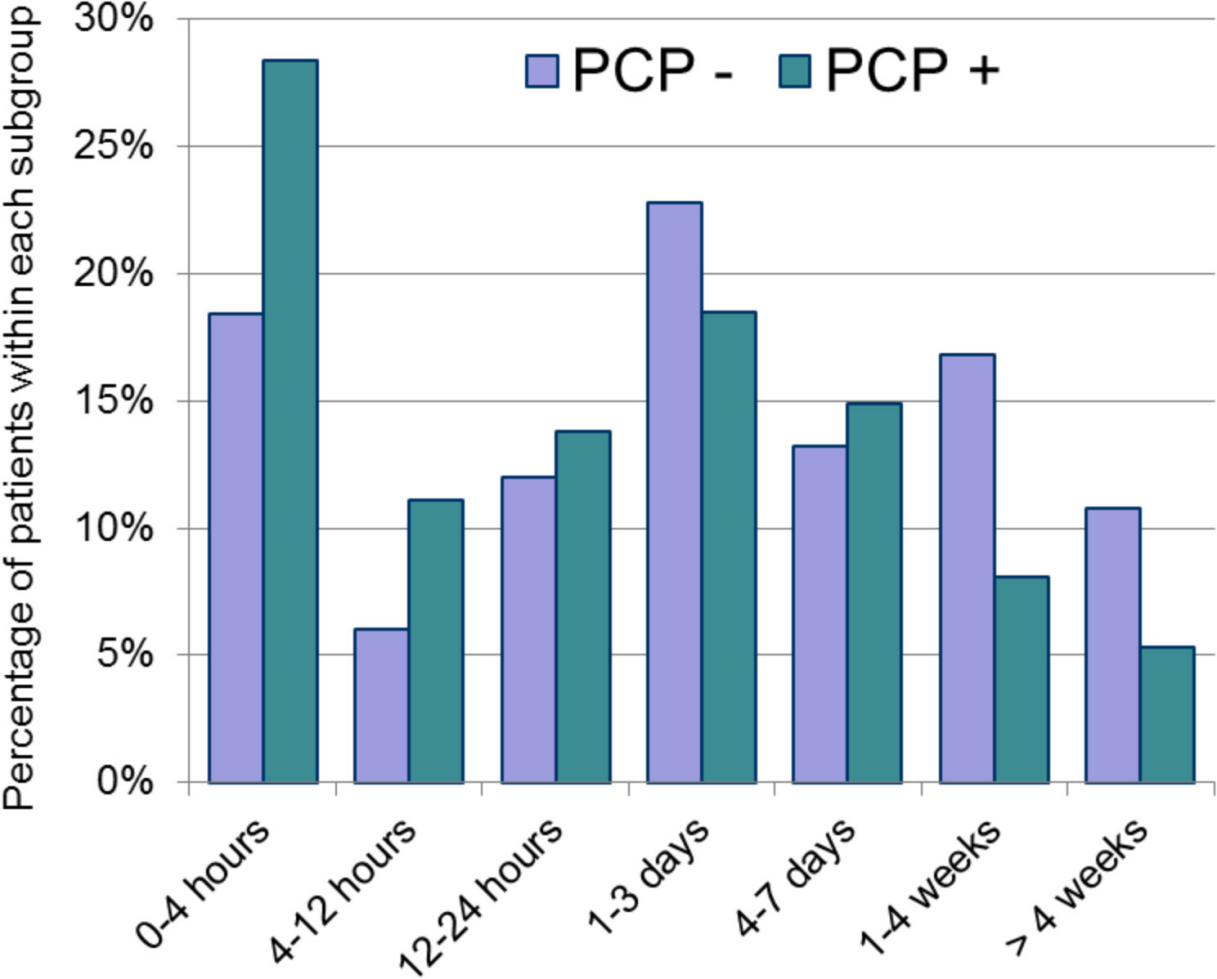
Duration of symptoms prior to presentation*. PCP-, Patient does not have a primary care provider (n = 250). PCP+, Patient does have a primary care provider (n = 2,134). * p<0.001.

The duration of symptoms prior to arrival to the emergency department is depicted in Fig 4; 27.3% of visits occurred within 4 hours and 51.4% within 24 hours of symptom onset. Patients without a primary care provider waited significantly longer before presenting (p<0.001 using linear-by-linear test of association). A significantly higher proportion of patients presenting within 4 hours had injuries (67.5%) compared to later timeframes (>4 hours), when illness-type complaints were more prevalent (70.9%, p<0.001).

**Fig 5:**
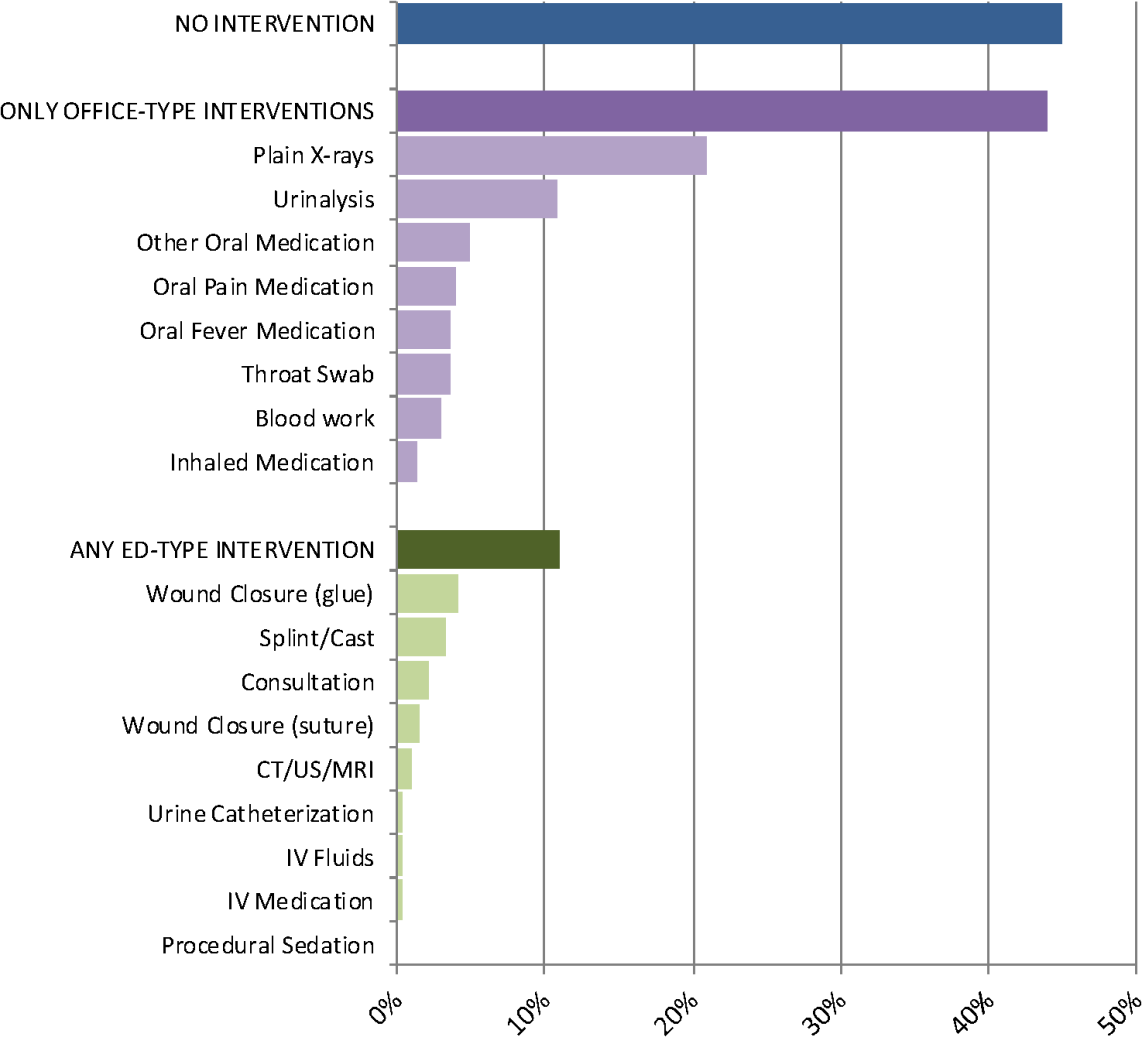
Interventions required during the emergency department visit.

Nearly all patients completed the visit and were discharged home (96.2%); 97 (3.5%) left without being seen and 9 (0.3%) patients were admitted. Forty-five percent of patients required no intervention beyond the history and physical exam completed by the physician or nurse practitioner (Fig 5). Further, 44% required only office-type interventions that a primary care provider could provide in our large urban environment (e.g., plain x-ray, urinalysis, throat swab), while the remaining 11% of patients required interventions that would merit an emergency department visit (e.g., wound closure, splinting or casting, consultation with a subspecialist).

**Fig 6:**
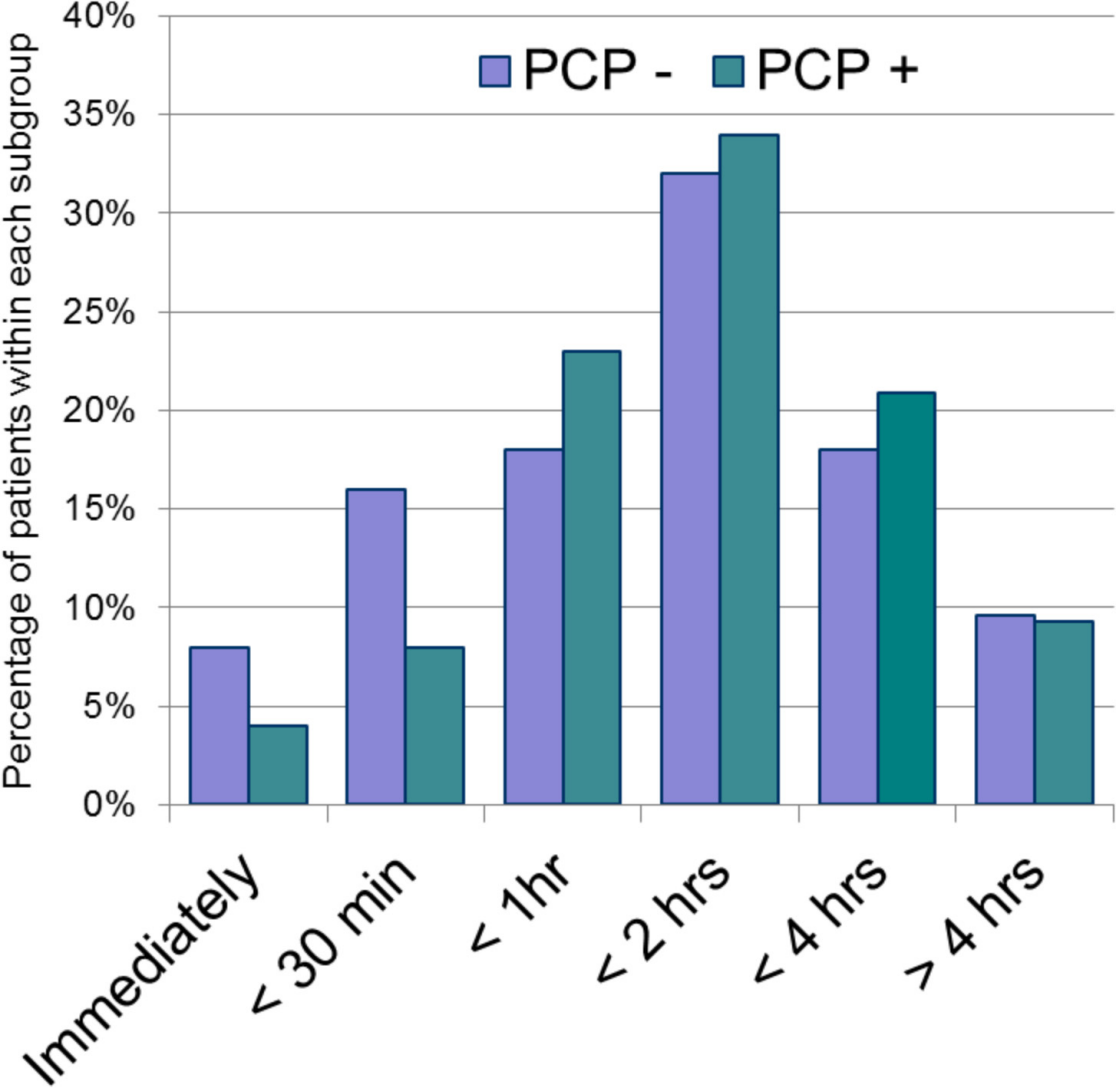
Parental sense of urgency to see the emergency physician*. PCP-, Patient does not have a primary care provider (n = 239). PCP+, Patient does have a primary care provider (n = 2,057). * *p*<0.02.

Parents were asked how soon after arrival they felt their child needed to be seen, reflecting their sense of urgency (Fig 6). This was high, with 36.1% expecting to be seen within 1 hour and 70.1% within 2 hours of arrival. Parents of patients without a PCP had a significantly higher sense of urgency (40.6% within 1 hour, compared to 35.6%, p<0.02 using linear-by-linear test of association).

**Fig 7:**
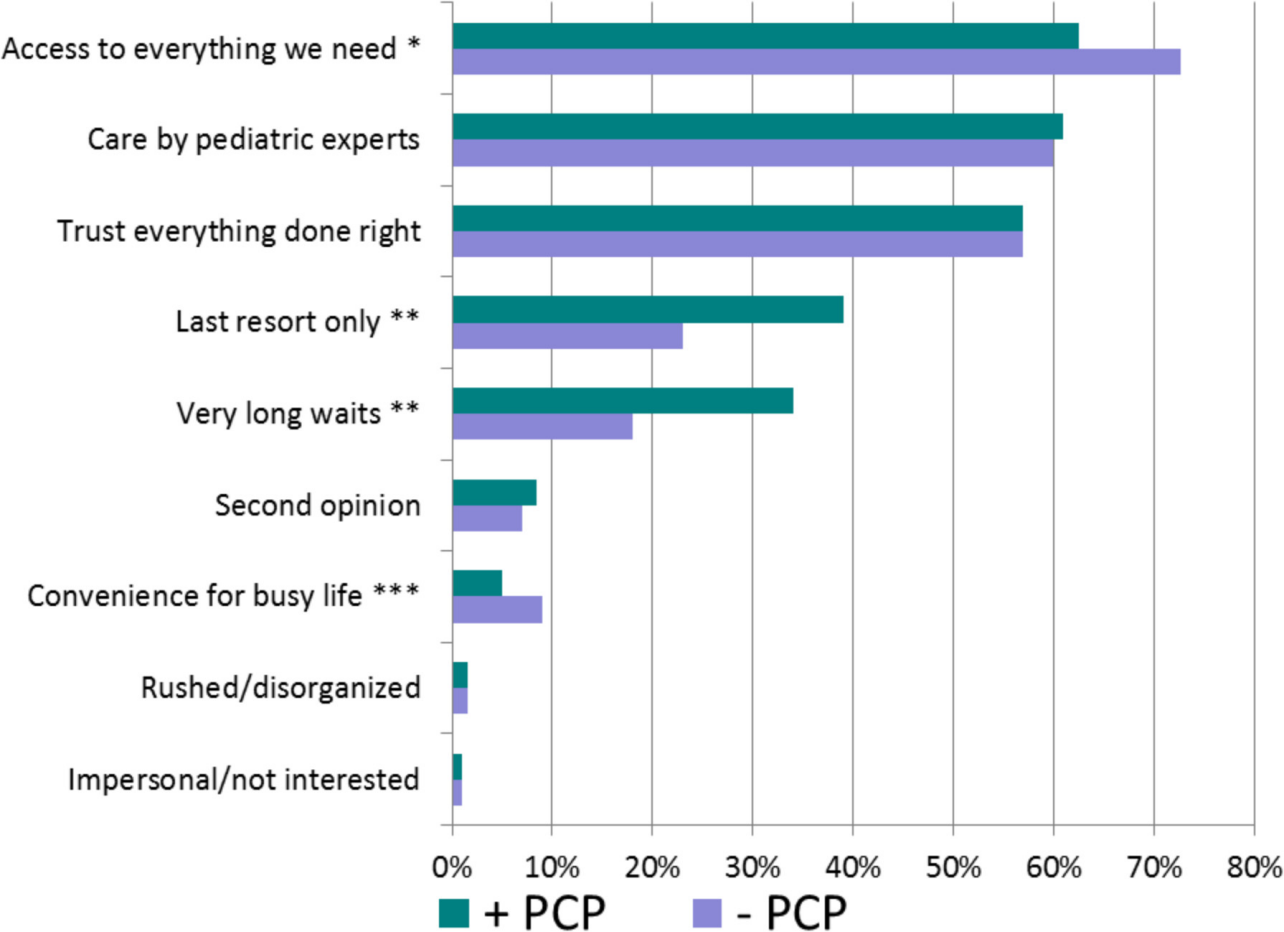
Motivations and perceptions about coming to the emergency department. PCP-, Patient does not have a primary care provider. PCP+, Patient does have a primary care provider p values represent significant differences between subgroups by Pearson’s chi-square test. * p = 0.001. ** p<0.001. *** p = 0.005.

Parents were asked to select from nine positive and negative statements regarding their general motivations and perceptions to seek care at the pediatric emergency department, not specific to this visit (Fig 7). Most selections were positive with high numbers of respondents indicating that the pediatric emergency department provided access to everything their child needed for care (63.6%), access to pediatric experts (60.0%), and trust that things will be done correctly (57.0%).

Parents reported alternate ways they tried to access care for their child before coming to the pediatric emergency department. Thirty-six percent reported not seeking alternative options because they believed the pediatric emergency department was the most appropriate place for their child’s problem; 4.7% of respondents reported not being aware of other options. Twenty-five percent of respondents reported that they had attempted to obtain an acute appointment with their primary care provider, 20.7% used a telephone information line for advice, and 18.5% tried to access a walk-in or urgent care clinic. Amongst patients with a primary care provider, more parents presenting during weekday office hours had attempted to reach their PCP (39.3%) than those presenting at other times during the week when the provider’s office was likely closed (24.3%, p<0.001).

**Fig 8:**
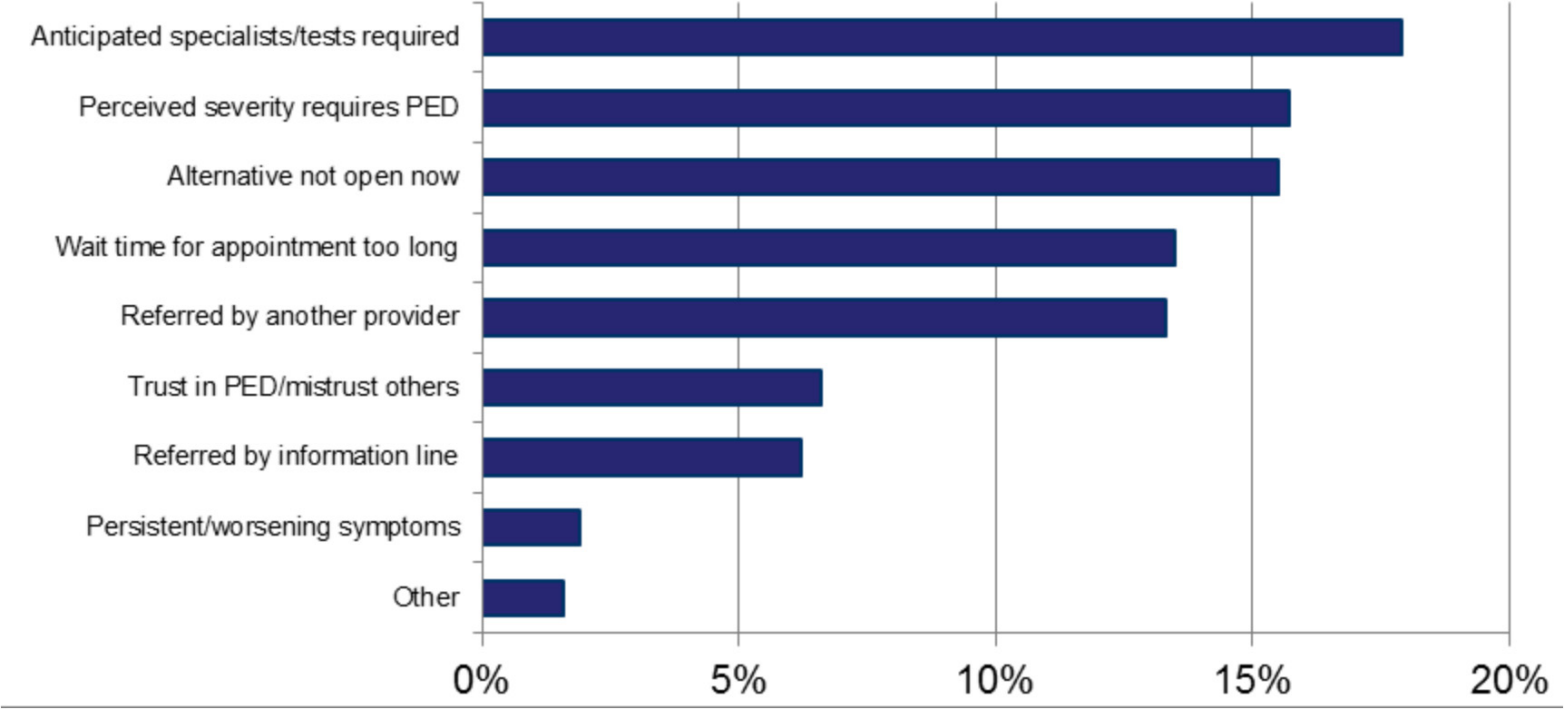
Themes of qualitative responses on why alternative options did not serve their needs. PED, Pediatric Emergency Department.

Qualitative responses were analyzed for why these options did not serve their needs; a summary of themes is depicted in Fig 8. In general, there was a strong sense that the child’s problem warranted the specialized services of the pediatric emergency department, or that an alternate practice setting was either not open or unable to see the child in an acceptable timeframe.

## Discussion

We examined the complex issues influencing increasing use of our pediatric emergency department for low-acuity problems. Responses to our parental survey were separated and compared on the basis of whether the child had a primary care provider. We showed that most patients in our region have a primary care provider, though significant differences occur between the two provinces we serve. The health care system in Quebec has struggled for many years with the lowest per-capita health spending of all Canadian provinces and second lowest per-capita spending on physicians[22] resulting in low primary care affiliation.

Despite a high rate of primary care affiliation among the study patients, a high proportion of visits occurred during office hours and within 24 hours of symptom onset. Parental responses suggested a high level of anxiety for their child’s presentation, supported by motivations to quickly obtain care by pediatric experts in a location that could provide everything their child might require. However, only slightly more than half of patients required any intervention, and most of those interventions could have been provided in an office environment. Finally, only one quarter of patients attempted to get an appointment with their child’s PCP; the office not being open or there not being an appointment within an acceptable time period was cited most often as why this option was not sufficient.

Our study supports that timely access to primary care providers for low-acuity problems is a key deficiency of our health care system, according to the respondents. This finding is similar to the primary care access barriers cited in several American studies[8,23–25]. In a 2013 international comparison survey, Canada was ranked worst for wait times to see a doctor or nurse when sick, with only 41% able to be seen the same or next day[26]. Having a primary care provider is not sufficient; ensuring timely access for acute problems is needed in order to decrease emergency department utilization[27–29]. Though we did not ask parents about their primary care provider’s availability, the low number that attempted to obtain an appointment leads us to postulate that parents assume their provider was not available, possibly from prior attempts to receive care in similar situations. In a systematic review of non-emergency department interventions to reduce emergency department utilization, the highest number of studies showing impact were for patient financial incentives and for managed care models (e.g. primary care physician capitation or gatekeeping)[30]. In Ontario, primary health care reform has imbedded after-hours availability into Family Health Networks (FHN) and Family Health Groups (FHG), with resultant lower emergency department utilization for patients in the FHN model compared to FHG and straight fee-for-service[31,32]. Just under one third of our respondents identified their provider as being part of one of these models. Health planners and policy makers should expand the number of physicians participating in the FHN model to further reduce emergency department visits for low acuity problems. Additional health reforms successfully implemented in other jurisdictions could include open access scheduling in primary care offices[33], collaborative partnerships with primary care providers to develop new non-emergency department options for urgent care[34,35], and information provided to patients and their parents of their provider’s availability[36].

Our study also supports that parental sense of urgency may exacerbate the lack of timely access, leading them to forego any delay to see their primary care provider in favour of having their child’s problem assessed and managed quickly in a fully-resourced environment. This need for reassurance, belief that the primary care office lacks the tests and treatments necessary for their child’s condition, and trust in the medical expertise available at the pediatric emergency department, have also been highlighted in other studies[7,37–40]. As low health literacy has clear association with increased emergency department utilization[41], parental education could increase knowledge and confidence to manage common low-acuity problems with an appropriate ‘watch and wait’ approach[7]. Educational initiatives must also address perceptions that primary care providers are unable to provide the treatments a child might require. It must be recognized, however, that the impact of patient and parental education initiatives on emergency department use has been variable[30,42–44].

Our results are strengthened by the large sample size and high survey response rate. Determination of PaedCTAS, and hence eligibility, was determined in the usual manner by triage nurses using this standard tool, which has been validated for conformity and inter-rater reliability in the Canadian pediatric emergency department context[45–48]. Finally, our pediatric emergency department serves two distinct patient populations with different rates of primary care affiliation, Ontario and Quebec, providing a unique opportunity to study these issues.

### Limitations

There were several limitations in this study. Choosing random weeks or random days over the course of the year could have reduced potential sampling bias. Further, only 78% of low-acuity patients were approached, likely due to this task being missed by registration clerks when facing long patient queues. We were unable to assess for other possible sampling biases introduced if certain sub-populations were over or under represented in responding (i.e., socioeconomic status, literacy level, distance travelled to receive care). This was a single centre study; results may not be generalizable to other pediatric emergency departments where the baseline rate of primary care affiliation is different, or where longer wait-times make the pediatric emergency department less appealing than other community-based options for low-acuity problems. Our survey tool was not validated, and parental anxiety and health literacy was not formally measured. Additionally, the survey tool did not address prior experience with our pediatric emergency department or look at revisit issues. The treating physician potentially introduced bias during collection of intervention data by under-reporting what patients required; this could have been reduced using a blinded research assistant to abstract this information. We made assumptions about which interventions could be reasonably provided in an office environment; plain x-ray was the most common, as required by the large proportion of low-acuity patients presenting with acute injuries, yet delay to radiology reporting or lack of physician skill interpreting pediatric x-rays may preclude this approach. Assigning plain x-ray to be an emergency department-type intervention would have significantly increased the proportion of patients necessitating an emergency department visit. Finally, we did not validate parent reports that their primary care provider was not available.

## Conclusion

Many low-acuity visits to our Canadian tertiary pediatric emergency department occur despite the child having a primary care provider. Parents report a lack of timely access to their provider while also having a high sense of urgency about their child’s problem, which contributes to them seeking care from the pediatric emergency department. Parents believe their child will require specialized tests or treatments only available at the emergency department, though this is rarely the case. Trust in pediatric experts may also contribute to over-utilization. Improved primary care access and educational initiatives for parents may help address the growing problem of low-acuity pediatric emergency department visits. Different initiatives will be needed to address the primary care challenges and infrastructures unique to the two provincial health system delivery models. These initiatives will need to be evaluated for efficacy and acceptance to those affected.

## Supporting Information

S1 File Survey Tool. (PDF)

S2 File Data Collection Form. (PDF)
