# Supplementary material for: Understanding Low-Acuity Visits to the Pediatric Emergency Department
Source: PLoS One. 2015 Jun 17;10(6):e0128927. doi: 10.1371/journal.pone.0128927 (PMC4471269; doi:10.1371/journal.pone.0128927)
Supplement: S1 File — (PDF) [file pone.0128927.s001.pdf]

Appendix 1: Survey Tool

SSurvey # ________

Understanding Low Acuity Visits to the Emergency Department

Your child has been seen by the Triage Nurse and found to have a problem that is considered by the Ministry of Health to be 'Low Acuity'. That means that your child's condition is stable and **may** have been safely and effectively managed by other providers in the community.

In an effort to provide better care, we want to understand your reasons for coming to the CHEO Emergency Department today. We also want to understand what other health care options you considered or attempted to access, prior to coming to the Emergency Department.

Please complete this brief survey while waiting to see the doctor or nurse practitioner. Please deposit it in one of the survey collection boxes throughout the Emergency Department. In addition to your survey responses, we would like to collect some basic information from your child's chart, such as which region you live in, how long you waited to be seen by the doctor, and what types of tests or treatments your child required for this visit.

Your participation is voluntary. Participating will not change the care your child receives today, or the length of time you have to wait. Your answers will be kept anonymous and only information about groups of patients will be reported.

By returning a completed survey, we will assume that you have consented to participate in both the survey and review of your child's chart. If you choose to **NOT** have your child's chart information included in the results, please check below before returning your survey.

- ☐ **Do NOT use my child's information in this study.**

Thank you!

For information about this survey, please contact:

Dr. Ken Farion
Acting Medical Director, ED
(613) 737-7600 x2318

Megan Wright
Operations Director, ED
(613) 737-7600 x2333

Appendix 1: Survey Tool

1. **Do you have a regular healthcare provider for your child?**

- ☐ Yes (Please continue to Question #2)
- ☐ No (Please skip to Question #4)

2. **Choose the type of regular healthcare provider that you have for your child:**

- ☐ Family Doctor
- ☐ Pediatrician
- ☐ Nurse Practitioner
- ☐ Walk-in or Urgent Care Clinic (first come, first served)
- ☐ Public Health Clinic/CLSC
- ☐ Naturopath, Chiropractor, or Homeopath
- ☐ Other:________________________

3. **If you selected *Family Doctor, Pediatrician or Nurse Practitioner* above, in what type of practice do they work? Otherwise, if you selected one of the other options, please skip to Question #4.**

- ☐ Independent office or small group practice
- ☐ Family Health Network or Family Health Team
- ☐ Unsure

4. **Which of the words or phrases below represents the CHEO Emergency Department for you? <u>Please choose all that apply</u>.**

- ☐ Access to everything my child needs for care
- ☐ Very long waits
- ☐ Convenience with my busy life
- ☐ Only as a last resort
- ☐ Opportunity for a second opinion
- ☐ Rushed and disorganized
- ☐ Trust that things will be done right
- ☐ Impersonal or not interested in my child
- ☐ Care by pediatric experts

5. **What is the main problem that brought you to the Emergency Department today?** (Please choose the **ONE** problem that was most concerning to you)

- ☐ Fever
- ☐ Vomiting and/or diarrhea
- ☐ Cough, stuffy nose or difficulty breathing
- ☐ Ear ache or sore throat
- ☐ Tummy pain or constipation
- ☐ Urinary (pee) symptoms or genital concerns
- ☐ Headache, dizziness, confusion
- ☐ Head injury or concussion
- ☐ Laceration or cut/scrape
- ☐ Broken bone, sprain or other limb/joint pain or injury
- ☐ Tooth or mouth injury, pain, or infection
- ☐ Diet or feeding problems
- ☐ Swallowed object or object in the nose, ear or skin
- ☐ Rash, bite or other skin condition
- ☐ Mental health (depression, anxiety) or parenting issue
- ☐ Other:________________________

Appendix 1: Survey Tool

6. How long has your child had the main symptom or problem that brought you to the Emergency Department today?

☐ 0-4 hours	☐ 12-24 hours	☐ 4-7 days
☐ 4-12 hours	☐ 1-3 days	☐ 1-4 weeks
		☐ More than 4 weeks

7. When you decided to come to the Emergency Department, how soon did you expect to be seen by a doctor for your child's symptom or problem?

☐ Immediately	☐ Within 1 hour	☐ Within 4 hours
☐ Within 30 minutes	☐ Within 2 hours	☐ More than 4 hours

8. Before coming to CHEO, what other ways did you try to get care for your child? <u>Please select all that apply.</u>

☐ Telehealth Ontario or Québec Info Santé line
☐ My regular healthcare provider office for an appointment
☐ My regular healthcare provider after-hours telephone advice line
☐ Walk-in/Urgent Care/Public Health Clinic/CLSC
☐ Another Emergency Department
☐ Other: ____

☐ I did not try another option because I thought the CHEO Emergency Department was the most appropriate place for care of my child's problem.

☐ I did not try another option because I did not know of any other options.

9. For each of the other ways you tried in question #6, please briefly describe why the option was not adequate to meet your child's needs.

____

____

____

10. Do you have any further comments about this topic or suggestions for making CHEO's Emergency Department better?

____

____

____

____
