# Supplementary material for: Understanding Low-Acuity Visits to the Pediatric Emergency Department
Source: PLoS One. 2015 Jun 17;10(6):e0128927. doi: 10.1371/journal.pone.0128927 (PMC4471269; doi:10.1371/journal.pone.0128927)
Supplement: S2 File — (PDF) [file pone.0128927.s002.pdf]

S2: Data Collection Form

DATA COLLECTION FORM
Understanding Low Acuity Visits to the Emergency Department

Assigned Survey #: |PLACE LABEL HERE|

1. Patient Age: ☐ 0-30 d ☐ 1-3 m ☐ 4-6 m ☐ 7-11 m
   ☐ 1-3 y ☐ 4-6 y ☐ 7-10 y ☐ 11-14y ☐ 15-18y
2. Postal Code: ___ ___ ___ (X0X if outside Canada or unknown)
3. Primary Care: ☐ None ☐ Other ☐ Indep FD ☐ Indep Ped ☐ FHT/FHN
4. Day of Arrival: ☐ Weekday (***Monday 08:00 – Friday 17:00***)
   ☐ Weekend (***Friday 17:00 – Monday 08:00 + Stats***)
5. Time of Arrival (T1): __ __ : __ __ (24-hour clock)
6. Time of MD Assessment: __ __ : __ __ ☐ Next day
7. Time of Discharge: __ __ : __ __ ☐ Next day

TO BE COMPLETED BY MD/NP/FELLOW/RESIDENT – PLEASE KEEP WITH CHART

8. Were any interventions performed? ☐ **No** ☐ **Yes** (***If yes, check all that apply***).

☐ **Oral fever control**	☐ **Urinalysis**
☐ **Oral pain control**	☐ **Urine catheterization**
☐ **Other oral medication**	☐ **Throat swab**
☐ **Inhaled medication**	☐ **Blood work/other labs**
☐ **Plain X-rays**	☐ **Intravenous fluids**
☐ **CT/US/MRI**	☐ **Intravenous medication**
☐ **Wound closure (glue/tapes)**	☐ **Splint or cast**
☐ **Wound closure (sutures)**	☐ **Procedural sedation**
☐ **Consultation**	☐ **Other** ___________

9. Diagnosis: ① ____
② ____

10. Disposition: ☐ **Discharged to home** ☐ **Admitted to CHEO** ☐ **LWBS**

11. Next Follow-up:
☐ **No specific F/U (RTER prn, F/U FD)**
☐ **See F/D in specific amount of time**
☐ **Return to ED at specific time (R/A, test, imaging)**
☐ **Referral to CHEO specialist or clinic**
☐ **Patient was admitted to the floor**
☐ **Patient LWBS**
